# Concurrent Validity of Zeno Instrumented Walkway and Video-Based Gait Features in Adults With Parkinson’s Disease

**DOI:** 10.1109/JTEHM.2022.3180231

**Published:** 2022-06-03

**Authors:** Andrea Sabo, Carolina Gorodetsky, Alfonso Fasano, Andrea Iaboni, Babak Taati

**Affiliations:** KITE Research Institute, Toronto Rehabilitation Institute—University Health Network (UHN) Toronto ON M5G 1L7 Canada; Institute of Biomedical Engineering, University of Toronto 7938 Toronto ON M5S 1A1 Canada; Division of NeurologyThe Hospital for Sick Children 7979 Toronto ON M5G 1X8 Canada; Edmond J. Safra Program in Parkinson’s Disease, Morton and Gloria Shulman Movement Disorders Clinic, Toronto Western HospitalUniversity Health Network (UHN) Toronto ON M5G 1L7 Canada; Division of NeurologyUniversity of Toronto 7938 Toronto ON M5S 1A1 Canada; Krembil Brain Institute Toronto ON M5T 1M8 Canada; Center for Advancing Neurotechnological Innovation to Application (CRANIA) Toronto ON M5G 2C4 Canada; Department of PsychiatryUniversity of Toronto 7938 Toronto ON M5S 1A1 Canada; Centre for Mental HealthUniversity Health Network (UHN) Toronto ON M5G 1L7 Canada; Department of Computer ScienceUniversity of Toronto 7938 Toronto ON M5S 1A1 Canada

**Keywords:** Computer vision, human pose-estimation, Parkinson’s disease, quantitative gait analysis, Zeno instrumented walkway

## Abstract

Background: Parkinson’s disease (PD) presents with motor symptoms such as bradykinesia, rigidity, and tremor that can affect gait. To monitor changes associated with disease progression or medication use, quantitative gait assessment is often performed during clinical visits. Conversely, vision-based solutions have been proposed for monitoring gait quality in non-clinical settings. Methods: We use three 2D human pose-estimation libraries (AlphaPose, Detectron, OpenPose) and one 3D library (ROMP) to calculate gait features from color video, and correlate them with those extracted by a Zeno instrumented walkway in older adults with PD. We calculate video-based gait features using a manual and automated heel-strike detection algorithm, and compare the correlations when the participants walk towards and away from the camera separately. Results: Based on analysis of 67 bidirectional walking bouts from 25 adults with PD, moderate to strong positive correlations were identified between the number of steps, cadence, as well as the mean and coefficient of variation of step width calculated from Zeno and video using 2D pose-estimation libraries. We noted that our automated heel-strike annotation method struggled to identify short steps. Conclusion: Gait features calculated from 2D joint trajectories are more strongly correlated with the Zeno than analogous gait features calculated from ROMP. Based on our analysis, videos processed with 2D pose-estimation libraries can be used for longitudinal gait monitoring in individuals with PD. Future work will seek to improve the prediction of gait features using a comprehensive machine learning model to predict gait features directly from color video without relying on intermediate extraction of joint trajectories.

## I. Introduction

Individuals with Parkinson’s disease (PD) experience deterioration of gait during the progression of their disease. Specifically, changes in step length, gait speed, axial rigidity, and rhythmicity have been observed as the disease progresses [1], [2]. In addition to disease progression, gait patterns in this population can fluctuate due to dopaminergic medications used to treat the symptoms of PD [1], [3].

Currently, quantitative gait assessment is commonly performed in the clinic using sophisticated motion-capture systems or instrumented walkways. However, these systems require large and specialized equipment that cannot be easily used outside of the clinic, thus requiring individuals with PD to visit specialized clinics to have their gait assessed. This has motivated the development and evaluation of various tools for measuring quantitative gait parameters outside of the clinic to facilitate more frequent and convenient monitoring of gait.

Body-worn inertial measurement units (IMUs) have been proposed as a means to capture gait information in this population [4]–[6]. Previous work has validated that body-worn IMUs can detect gait events such as heel-strike and toe-off in individuals with PD with high precision and recall [7]. Furthermore, spatial gait parameters of stride length, stride velocity, and vertical displacement of the shank have also been validated against an optical motion-capture system in a healthy adult population, achieving strong correlation (r >.9) [8]. However, while accurate and light-weight, there are challenges associated with the use of IMUs for longitudinal analysis of gait in older adults outside of the clinic. Multiple IMUs are often needed to capture gait information accurately, and compliance in people with PD is low (62 – 68%), even for systems consisting of a single wrist-worn device [9].

Vision-based systems provide an alternative to IMUs for unobtrusive gait assessment outside of the clinic. Gait features calculated using a Microsoft Kinect sensor, which consists of a depth and color video camera, have been validated against both gold-standard motion capture systems and GAITRite instrumented walkways (CIR Systems Inc., Franklin, NJ, USA) [10], [11]. While systems that use multiple Microsoft Kinect sensors may be well-suited for environments where researchers or technicians can set-up the devices appropriately, these systems are not feasible for in-home use by older adults. As a more accessible alternative, color video recorded from ubiquitous consumer-grade webcams or smartphone have been proposed for gait assessment in non-clinical settings. Recent advances in pose-estimation libraries allow for extraction of joint trajectories from consumer-grade color video [12]–[15]. Previous work has used videos from a single consumer-grade camera to successfully extract joint trajectories and predict parkinsonism severity [16], [17], and specifically on the gait item of the Unified Parkinson’s Disease Rating Scale (UPDRS-gait) using machine learning models [18]–[21]. Furthermore, spatial-temporal and balance gait features extracted from smartphone videos have been correlated with those obtained from an IMU system in healthy older adults [22]. However, gait features extracted from standard color video have not yet been validated against clinical gait assessment systems in older adults with PD.

This work seeks to validate whether gait features calculated from standard color video using joint trajectories extracted using three open-source pose-estimation libraries (AlphaPose, Detectron, OpenPose) can be used to assess gait in individuals with PD. To achieve this goal, the video-based gait features will be correlated with the corresponding gait features extracted from a Zeno instrumented walkway (ZenoMetrics, Peekskill, NY, USA) and PKMAS - ProtoKinetics Movement Analysis Software (ProtoKinetics LLC, Havertown, PA, USA). The Zeno instrumented walkway and PKMAS system are commonly used for clinical gait assessment and have been previously validated for use in individuals with PD [23], [24].

As the calculation of gait features from video is dependent on the detection of heel-strikes, an automated algorithm is compared with manual annotations of heel-strikes. Finally, the correlation of gait features obtained from the two systems is examined separately for walking bouts for which the participant is walking towards and away from the camera. This work serves as a first step towards understanding the capabilities and limitations of quantitative gait assessment of individuals with PD using a single-consumer grade camera.

## II. Methods

### A. Data Collection

**FIGURE 1.**
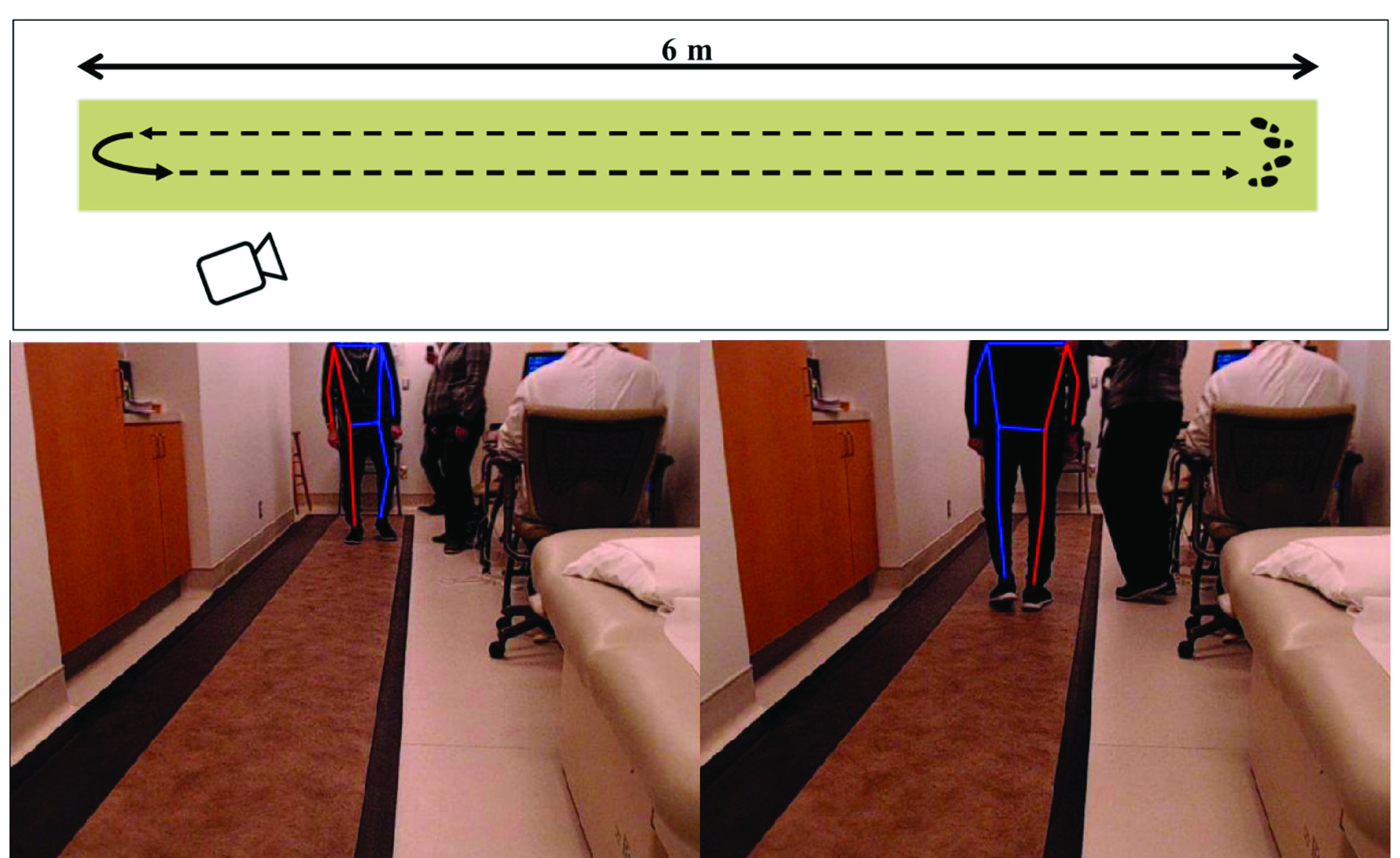
The top panel presents an overhead view of data collection environment. Participants were instructed to walk along a 6 m instrumented walkway while simultaneously being recorded by a standard color camera. The bottom panels show the major joints detected by the OpenPose pose-estimation library as a participant walks toward (left) and away from (right) the camera.

Participants with a diagnosis of idiopathic PD were recruited for this study. Participants were assessed in an off treatment state and while on their regular therapy, which in some cases also included deep brain stimulation (DBS). When possible, participants were assessed under multiple treatment conditions during the same clinical visit, and all walks were analyzed in this study. Walks were assessed on the new version of the UPDRS (MDS-UPDRS) from the video recordings by a specialist clinician (neurologist) affiliated with the study [25]. The research ethics board of the institute approved the study protocol. Participants were instructed to walk 6 m along a Zeno^™^ Walkway Gait Analysis System (Zeno, 120 Hz sampling frequency), turn around at the end of the walkway, and then walk 6 m back to the starting position (Fig. 1). A tripod mounted camera (Logitech C920, 78° field of view, recording at 480 × 640 resolution, 30 Hz) was used to record color video of each walking bout. The PKMAS suite was used to synchronize and simultaneously record both video and data from the Zeno. The camera was positioned near one end of the instrumented walkway (Fig. 1) and was not moved between trials recorded on the same day, but small changes in camera position and orientation occurred between data collection days. The participants were evaluated in the clothes they wore to the clinic and the standard ceiling lights were used to illuminate the room (Fig 1).

### B. Calculation of Gait Features

Parameters of gait were extracted independently from the data collected by the Zeno (using the PKMAS suite) and from the color videos. Due to the position of and field of view of the camera, the participants’ ankles were not visible in the video frame when they were close to the camera. The videos were thus first manually segmented to exclude the sections where the ankles were not visible, as well when the participant was turning or stationary at the beginning and end of each recording. No exclusion of bouts by step or walk quality (e.g., due to shuffling gait) was performed. The first and last steps were retained in each walking bout as the planned analysis sought to correlate features from video and Zeno, rather than calculate exact values during constant walking speed. Each walking bout was divided into two walking segments: one where the participant was walking towards the camera and one where the participant was walking away. The same criteria were applied to filter the steps detected by the Zeno-PKMAS system, ensuring that the same steps were selected for analysis by both modalities.

#### 1) 2D Gait Features From Video

Three open-source human pose-estimation libraries (AlphaPose, Detectron, OpenPose) were used to extract the positions of body joints from the color videos [12]–[15]. These libraries use pretrained deep learning models to predict the $x$ and $y$ pixel coordinates of major body joints in each frame of the video. Confidence scores representing the model’s certainty of its prediction for each joint are also output by the models. In our experiments using an RTX 2080Ti GPU, the average inference frames per second (FPS) was 12.8 for AlphaPose, 4.4 for Detectron, and 54.9 for OpenPose.

The predicted joint positions were combined across frames to create trajectories of joint positions over time. Joint positions with low confidence scores were removed and interpolated using adjacent timesteps. As the confidence scores output by the three pose-estimation libraries are not calibrated, the threshold denoting “low” confidence varied (0.50 for AlphaPose, 0.15 for Detectron, and 0.65 for OpenPose), but was selected such that on average less than 10% of the timesteps were interpolated for each joint in each pose-estimation library. Finally, a zero-phase, second-order low-pass Butterworth filter with an 8 Hz cut-off was used to smooth the joint trajectories to remove noise and jitter.

**FIGURE 2.**
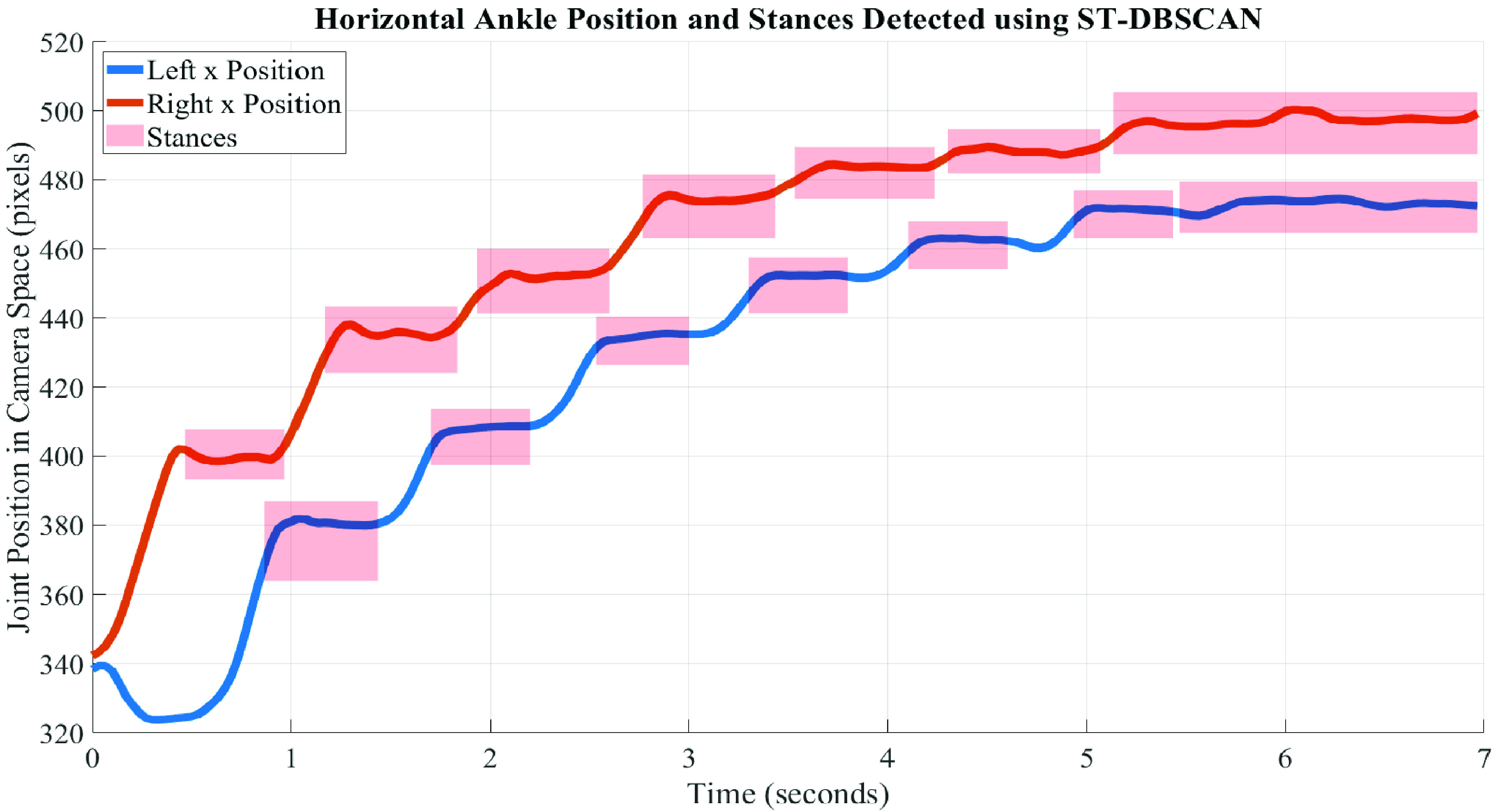
Horizontal ankle positions for a sample walk of a participant walking away from the camera. The stances detected by the ST-DBSCAN algorithm are denoted by red boxes, and heel-strikes were selected as the first timestep of each detected stance. Note that as the participant walks farther from the camera (after ~5 seconds), the resolution of the joint trajectory signal is insufficient for the ST-DBSCAN algorithm to identify the last step (instead it is combined into the previous stance).

As a first step toward calculating gait features from video, heel-strike events were identified using two methods: automatically using a clustering algorithm and through manual annotation. To automatically identify heel strikes, the spatial-temporal density-based spatial clustering of applications with noise (ST-DBSCAN) algorithm was used [26]. This algorithm deterministically groups data points into clusters that are close in both space and time. In the context of gait analysis, this algorithm can be used to identify stances when the position of each ankle keypoint remains stationary (on the ground) for a specified period of time [27]. After identifying the stances in each ankle trajectory, heel-strikes were denoted as the first timesteps of each detected stance. Fig. 2 presents the joint trajectories of the ankles in the horizonal (x) direction for a sample walk from the dataset. The stances detected by the ST-DBSCAN algorithm are denoted by red boxes, and heel-strikes were extracted as the first timestep of each stance. Fig. 2 only presents the horizontal trajectories to improve visual clarity, but both spatial dimensions ( $x$ and $y$) in camera space were used to cluster the data and identify stances. As a comparison to the ST-DBSCAN method, heel-strikes were also manually annotated directly from the videos.

The joint trajectories and heel-strike labels were used to calculate five features of gait for each walking bout: cadence, number of steps, average step width, and the coefficient of variation (CV) of step width and time. Two feature sets were computed, one using the ST-DBSCAN heel-strike annotations and the other using manual annotations. A comprehensive description of these gait features and how they were calculated is presented in previous work [28]. It is important to note that real-world distance measures cannot be calculated from the positions of joint coordinates in camera space. Instead, distance measures (such as step width) were normalized by the hip width of the participant in each frame to compensate for the distance of the participant from the camera. For this reason, spatial measures calculated from video have no associated real-world units.

#### 2) 3D Gait Features From Video

As a comparison to the three 2D pose-estimation libraries, ROMP, a state-of-the-art 3D pose-estimation library operating on monocular video was also explored as a means of extracting joint trajectories from video [22]. The average inference FPS was 31.2 for ROMP. This pose-estimation library also predicts the location of each joint in the depth ( $z$) direction. Seven gait features were calculated from this data: the five aforementioned features calculated from the 2D data, as well as two additional features of gait speed and step length that require data in the depth dimension.

#### 3) Gait Features From Zeno

Gait features were extracted for the same steps in the same walking bouts using the data collected by the Zeno walkway. Calculation of gait features from the data recorded by the Zeno instrumented mat was performed using the PKMAS software suite. Although more gait features are available from PKMAS, the ones selected for evaluation in this study were: the number of steps, cadence, velocity, step length, the mean and coefficient of variation (CV) of stride width, and the CV of step and swing time. As the distance measures calculated from video were unitless and normalized, the stride width obtained from the Zeno was analogously divided by the foot length. In subsequent sections of this manuscript, stride width refers to this normalized metric.

### C. Correlation Analysis

A correlation analysis was performed between pairs of gait features extracted from video and using the Zeno system. The pairs of gait features correlated from each feature set were selected through discussion with a neurologist specializing in movement disorders and are presented in Table 1. These gait features were chosen as they were determined to be most relevant clinically. Individuals with PD have decreased velocity, reduced stride and step time, decreased swing time, increased stride time, stride time variability and dual support time. These gait characteristics correlate with clinical progression of PD and recent systematic review showed that these are among the most commonly assessed features in gait assessments in individuals with PD [29].

**TABLE 1:** Paired Video and Zeno Gait Features for Correlation Analysis

Video Gait Feature – Zeno Gait Feature
Number of steps – Number of steps
Cadence – Cadence
Step width – mean – Stride width – mean
Step width – CV^*^ – Stride width CV
Step time – CV^*^ – Step time CV
Step time – CV^*^ – Swing time CV
Speed^**^ – Velocity
Step length – mean^**^ – Step length – mean

^*^ CV = coefficient of variation,

^**^ 3D gait features from ROMP only

The correlation analysis was performed independently for the video feature sets extracted using automated (ST-DBSCAN) and manual heel-strike annotation, and for each pose-estimation library. Furthermore, the subsets of bouts where the participants were walking towards or away from the camera were analyzed separately.

The D’Agostino-Pearson test was first used to check for normality of each of the video and Zeno features. Tables A and B of the Supplemental Material present the p-values for this normality test. At an alpha level of.05, most feature sets were not normally distributed, so Spearman’s rank correlation analysis was performed between each gait feature pair. Right-tailed p-values were calculated to assess whether the correlation coefficient was greater than 0 indicating positive correlation between Zeno and video features. Bonferroni correction was used to adjust for the number of comparisons with each Zeno gait feature.

Furthermore, a Wilcoxon signed-rank test was used to assess whether there was a significant difference between gait features in different medication and DBS treatment states. Further details are presented in the Supplemental Material.

All statistical analysis was performed using MATLAB 9.9.0 (2020, The MathWorks Inc., Natick, MA, USA). An external open-source package was used to perform the normality tests [30].

## III. Results

A total of 67 walking assessments from 25 participants were analyzed in this study. Table 2 presents demographic and clinical data of the study participants and walking bouts. Eleven participants only had one gait assessment, while fourteen had 4 associated data recordings each. In most cases, although not for all, these 14 participants were recorded twice (the first in an off medication and DBS state, and the second on medication and DBS) during each of two clinical visits.

**TABLE 2:** Clinical Characteristics of Study Participants and Walking Bouts

Study participants (n = 25)	
Age (years)	65.1 ± 7.3
Sex (% male, n)	64%, 16 male
PD duration (years)	11.6 ± 3.7
MDS-UPDRS part III total score – median (range)	41(17 – 83)
Walking bouts (n = 67)	
MDS-UPDRS part III, gait sub score – median (range)	1 (0 – 3)
DBS state during walking bout (% DBS on state)	36.9% (2 unknown)
Medication state during walking bout (% on medication)	43.3%

MDS-UPDRS = Movement Disorders Society revision of Unified Parkinson’s Disease Rating Scale, DBS = Deep brain stimulation

### A. Correlation of Zeno and Gait Features From 2D Pose-Estimation Libraries

The 67 recorded videos were divided into 134 walking bouts in which the participants were continuous ambulating. The participants were walking toward the camera in half of the walks and away from the camera in the other half. Table 3 presents the number of walking bouts for which video-based gait features were extracted in each direction using each 2D pose-estimation library. Note that a minimum of three detected steps for which joint trajectory data was available was necessary to calculate gait features from video. Differences in the number of walks for which gait features could be extracted from video is therefore dependent on both the heel-strike detection algorithm, as well as the presence of the underlying joint trajectory data. If the underlying joint trajectory data was not extracted successfully, the manual annotations could not be used as they did not correspond to any spatial data.

**TABLE 3:** Number of Walking Bouts With Successfully Extracted Gait Features Per Pose-Estimation Library and Direction of Walk

		Number of walks with successfully extracted video gait features	
		ST-DBSCAN heelstrike annotations	Manual heel-strike annotations
AlphaPose	Towards	66	64
	Away	49	58
Detectron	Towards	65	58
	Away	61	64
OpenPose	Towards	64	58
	Away	37	63

Gait features from the Zeno were successfully extracted for 133 bouts (the returning phase of one bout was not recorded). Based on the heel-to-heel measurements between the first and last step (as measured by the Zeno), the average length per walking bout was 329.0 ± 58.6 cm (normally distributed at p <.05).

The correlation between each pair of video and Zeno gait features was evaluated on all walking bouts. Table 4 presents Spearman’s rank correlation coefficient (rho) and the Bonferroni-adjusted $p$-value from a right-tailed hypothesis test evaluating statistical significance of the correlation being positive. A total of 108 correlations are presented in Table 4, and a Bonferroni correction factor of 18 was used to account for multiple comparisons within each pair of gait features.

**TABLE 4:** Spearman’s Rank Correlation Coefficient (Rho) and Bonferroni-Adjusted Right-Tailed p-Values for Correlation Between all Walking Bouts

			ST-DBSCAN						Manual					
			Away from Camera		Towards Camera		Both		Away from Camera		Towards Camera		Both	
Video gait feature	Zeno gait feature	Detector	rho	$^{{p}}$	rho	$^{{p}}$	rho	$^{{p}}$	rho	$^{{p}}$	rho	$^{{p}}$	rho	$^{{p}}$
Number of steps	Number of steps	AlphaPose	.606	<.001	.627	<.001	.621	<.001	.785	<.001	.836	<.001	.801	<.001
		Detectron	.487	<.001	.593	<.001	.553	<.001	.785	<.001	.842	<.001	.804	<.001
		OpenPose	.674	<.001	.541	<.001	.594	<.001	.779	<.001	.855	<.001	.808	<.001
Cadence	Cadence	AlphaPose	.940	<.001	.695	<.001	.744	<.001	.923	<.001	.865	<.001	.893	<.001
		Detectron	.820	<.001	.545	<.001	.643	<.001	.923	<.001	.862	<.001	.892	<.001
		OpenPose	.930	<.001	.692	<.001	.735	<.001	.920	<.001	.862	<.001	.891	<.001
Step width – mean	Stride width – mean	AlphaPose	.341	.163	.601	<.001	.500	<.001	.393	.029<	.617	<.001	.514	<.001
		Detectron	.478	.001	.562	<.001	.521	<.001	.553	.001	.557	<.001	.550	<.001
		OpenPose	.339	.369	.585	<.001	.487	<.001	.160	1.000	.715	<.001	.450	<.001
Step width – CV	Stride width – CV	AlphaPose	.691	<.001	.403	.008<	.546	<.001	.580	<.001	.446	.005	.519	<.001
		Detectron	.585	<.001	.500	<.001	.561	<.001	.538	<.001	.456	.003	.524	<.001
		OpenPose	.615	<.001	.442	.003	.528	<.001	.501	.002	.505	<.001	.518	<.001
Step time - CV	Step time - CV	AlphaPose	.333	.191	.110	1.000	.186	.424	.432	.004	.608	<.001	.514	<.001
		Detectron	.408	.011	.106	1.000	.202	.213	.432	.004	.608	<.001	.514	<.001
		OpenPose	.282	.823	.172	1.000	.247	.115	.430	.005	.628	<.001	.524	<.001
Step time - CV	Swing time - CV	AlphaPose	.003	1.000	-.039	1.000	-.001	1.000	.407	.009	.469	.002	.421	<.001
		Detectron	.185	1.000	-.003	1.000	.063	1.000	.407	.009	.469	.002	.421	<.001
		OpenPose	.080	1.000	.074	1.000	.147	1.000	.409	.009	.494	.001	.435	<.001

**FIGURE 3.**
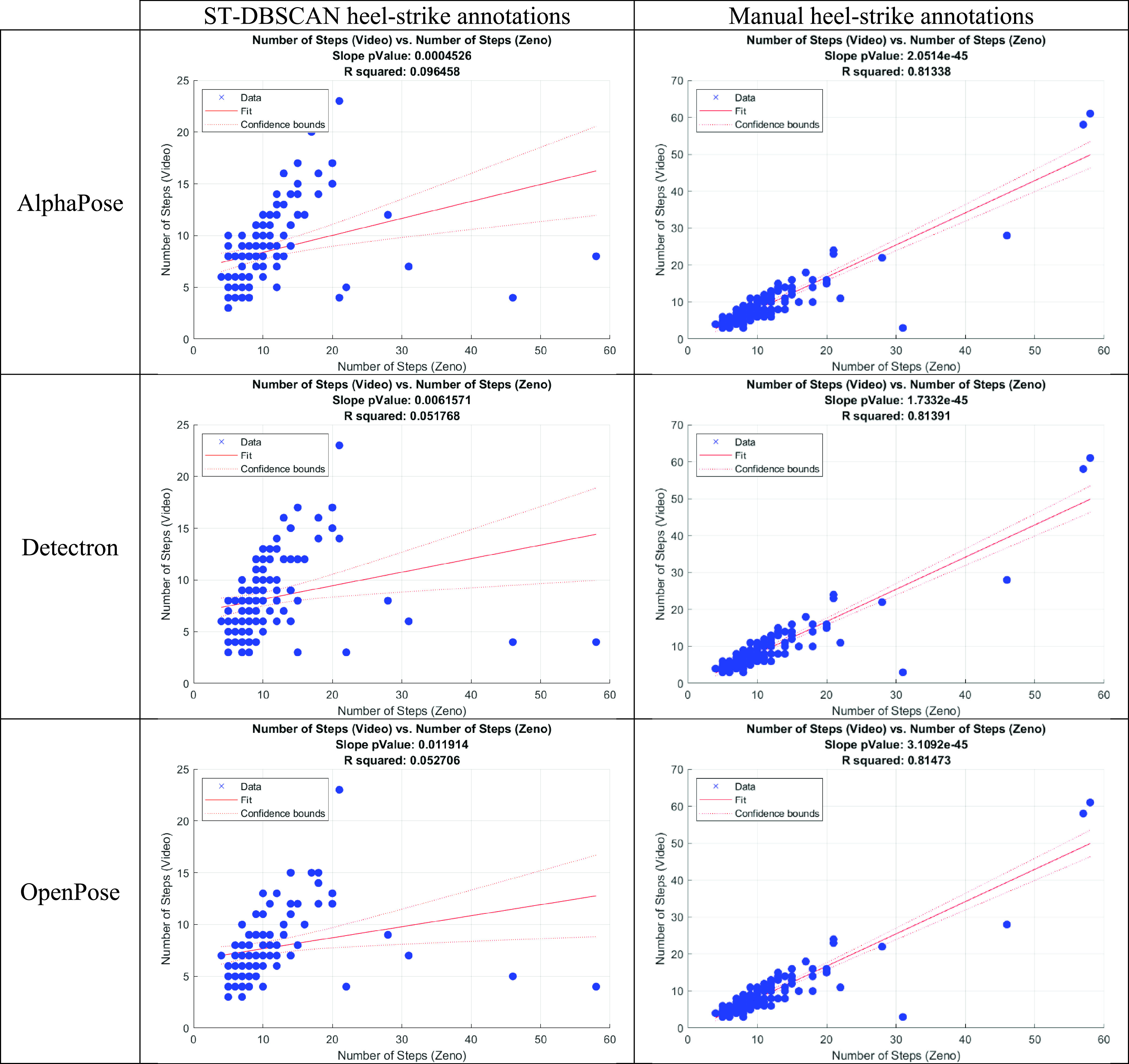
Scatterplots of number of steps detected by Zeno and video analysis, grouped by heel-strike annotation method. The red line represents the fit and confidence bounds of the linear regression model between the two variables.

From Table 4, it was observed that the rank correlation coefficient for the number of steps was significantly lower for the automatic ST-DBSCAN heel-strike annotation than for the manual annotation. To further investigate this difference in the correlation between the number of steps detected by the Zeno and number of steps detected by each heel strike annotation method from video, the scatterplots presented in Fig. 3 were created. From the left column of Fig. 3, it is observed that the ST-DBSCAN method fails to detect heel-strikes when the number of steps in the walk detected by the Zeno is large. As the ambulation distance was limited to an upper limit of 6 m for each walking bout, a large number of steps indicates that the participant was taking very short steps. Therefore, the ST-DBSCAN method is unable to identify heel-strikes when the participant takes many short steps. This is of concern as our method for calculating gait features relies on accurate heel-strike detection, so including walks for which the ST-DBSCAN method is known to be inaccurate affects all other correlations by introducing errors in the features estimated from video.

To determine the maximum number of steps for which the ST-DBSCAN method can accurately identify the number of steps in each walking bout in this dataset, the cut-off for the maximum number of steps as detected by the Zeno was varied and the correlation of the walks included under this threshold was examined. Fig. A of the Supplemental Material displays the R^2^ and slope of the linear regression model between the number of steps detected by the Zeno and vision-based ST-DBSCAN heel-strike detection method.

Based on the results in Fig. A, the strongest correlation was observed when the maximum number of steps per walk was approximately 20, and the correlation declines quickly with walks with greater than 20 steps. Walks with more than 20 steps were excluded and the correlation analysis was repeated. A total of 8 out of 133 walking bouts (less than 7% of bouts across all libraries) were excluded under the 20-step threshold. The mean step length (±SD) of the excluded walks was 10.8 ± 5.4 cm (range: 4.8 – 19.2 cm).

Table 5 presents the rank correlation coefficients and their Bonferroni-adjusted right-tailed $p$-value between all pairs of gait features calculated from video and Zeno while only including walks with up to 20 steps. A significantly stronger correlation between the number of steps detected by the ST-DBSCAN algorithm on video and the Zeno was observed when a 20-step cut-off was used. Significant positive correlations are observed among all gait feature pairs when manual annotation was used, but for automatic ST-DBSCAN heel-strike annotation, the correlation between step/stride time CV was not significant for most detectors and directions of walk.

**TABLE 5:** Spearman’s Rank Correlation Coefficient (Rho) and Bonferroni-Adjusted Right-Tailed p-Values for Correlation Between Walking Bouts With a Maximum of 20 Steps

			ST-DBSCAN						Manual					
			Away from Camera		Towards Camera		Both		Away from Camera		Towards Camera		Both	
Video gait feature	Zeno gait feature	Detector	rho	$^{{p}}$	rho	$^{{p}}$	rho	$^{{p}}$	rho	$^{{p}}$	rho	$^{{p}}$	rho	$^{{p}}$
Number of steps	Number of steps	AlphaPose	.770	<.001	.732	<.001	.750	<.001	.856	<.001	.788	<.001	.814	<.001
		Detectron	.500	<.001	.815	<.001	.670	<.001	.856	<.001	.796	<.001	.817	<.001
		OpenPose	.717	<.001	.717	<.001	.722	<.001	.850	<.001	.812	<.001	.823	<.001
Cadence	Cadence	AlphaPose	.933	<.001	.655	<.001	.713	<.001	.915	<.001	.846	<.001	.883	<.001
		Detectron	.801	<.001	.547	<.001	.619	<.001	.915	<.001	.842	<.001	.882	<.001
		OpenPose	.926	<.001	.654	<.001	.713	<.001	.911	<.001	.842	<.001	.880	<.001
Step width – mean	Stride width – mean	AlphaPose	.426	.031	.563	<.001	.511	<.001	.463	.006	.583	<.001	.530	<.001
		Detectron	.539	<.001	.523	<.001	.530	<.001	.614	<.001	.514	<.001	.562	<.001
		OpenPose	.427	.090	.562	<.001	.502	<.001	.228	1.000	.680	<.001	.460	<.001
Step width - CV	Stride width – CV	AlphaPose	.687	<.001	.396	.015	.542	<.001	.555	<.001	.447	.008	.508	<.001
		Detectron	.573	<.001	.501	<.001	.552	<.001	.509	<.001	.463	.005	.514	<.001
		OpenPose	.628	<.001	.424	.008	.519	<.001	.479	.006	.516	<.001	.507	<.001
Step time - CV	Step time – CV	AlphaPose	.244	.921	-.013	1.000	.085	1.000	.364	.041	.546	<.001	.441	<.001
		Detectron	.399	.017	.033	1.000	.175	.528	.364	.041	.546	<.001	.441	<.001
		OpenPose	.220	1.000	.043	1.000	.156	1.000	.362	.048	.569	<.001	.452	<.001
Step time - CV	Swing time - CV	AlphaPose	.011	1.000	-.164	1.000	-.064	1.000	.412	.011	.370	.066	.372	<.001
		Detectron	.265	.403	-.077	1.000	.074	1.000	.412	.011	.370	.066	.372	<.001
		OpenPose	.173	1.000	-.059	1.000	.115	1.000	.414	.011	.400	.032	.389	<.001

### B. Correlation of Zeno and Gait Features from 3D Pose-Estimation Library

The correlation analysis was repeated between Zeno and the gait features extracted using joint trajectories extracted using ROMP, the 3D pose-estimation library, for walks with a maximum of 20 steps. A total of 48 feature pairs were correlated and a Bonferroni adjustment factor of 6 was used to adjust for multiple comparisons between each feature pair. The results of this analysis are presented in Table 6.

**TABLE 6:** Spearman’s Rank Correlation Coefficient and Bonferroni-Adjusted Right-Tailed p-Values for Correlation Between Walking Bouts With a Maximum of 20 Steps Using the Romp 3d Pose-Estimation Library

		ST-DBSCAN						Manual					
		Away from Camera		Towards Camera			Both	Away from Camera		Towards Camera		Both	
Video gait feature	Zeno gait feature	rho	$^{{p}}$	rho	$^{{p}}$	rho	${P}$	rho	$^{{p}}$	rho	$^{{p}}$	rho	$^{{p}}$
Number of steps	Number of steps	.280	.196	.696	<.001	.522	<.001	.655	<.001	.744	<.001	.712	<.001
Cadence	Cadence	-.047	1.000	-.183	1.000	-.123	1.000	.906	<.001	.840	<.001	.864	<.001
Speed	Velocity	.075	1.000	.096	1.000	.090	1.000	.232	.451	.119	1.000	.172	.306
Step width - mean	Stride width - mean	.138	1.000	.188	.572	.150	.450	.551	<.001	.603	<.001	.567	<.001
Step length - mean	Step length - mean	.225	.428	.225	.346	.210	.126	.206	.605	-.370	1.000	-.104	1.000
Step width - CV	Stride width - CV	.301	.142	.006	1.000	.137	.561	.314	.148	.308	.080	.326	.005
Step time - CV	Step time - CV	.050	1.000	.101	1.000	.070	1.000	.281	.236	.299	.100	.297	.013
Step time - CV	Swing time - CV	.036	1.000	.340	.048	.197	.173	.361	.068	.093	1.000	.219	.111

For gait feature pairs that were also compared in the 2D analysis (Table 5), the correlations when the 3D ROMP library was used (Table 6) were generally weaker, particularly for the automated heel-strike method. Furthermore, although gait features that rely on data from the depth dimension (such as speed and step length) were analyzed between video (with ROMP) and Zeno, the correlations were not significant for these pairs.

A post-hoc analysis comparing the mean, mean absolute, and percent difference between the directly comparable gait measures of number of steps, cadence, and step time CV estimated from the Zeno and video are presented in Table C of the Supplemental Material. This analysis confirmed that the features extracted using the ROMP library had a significantly larger margin of error than any of the 2D pose-estimation libraries for these three directly comparable features.

### C. Comparison of Gait Features by Treatment State

A subset of bouts in this data set were collected during the same clinical visit, but under different medication and DBS treatments. The Supplemental Material presents an analysis examining whether the gait features of paired walking bouts are significantly different when collected under ON and OFF treatment states.

From Table D of the Supplemental Material, it can be noted that gait features calculated using manual step annotation were more consistently different when measured in ON and OFF treatment states. Differences between ON and OFF treatment states were also more consistently different when both directions of walk (towards and away from the camera) were analyzed.

## IV. Discussion

In this work, Spearman’s rank correlation coefficient between pairs of gait features extracted independently from a Zeno instrumented walkway and from standard color video was examined. Three 2D pose-estimation libraries (AlphaPose, Detectron, OpenPose) were the focus of the correlation analysis, however, this analysis was also repeated with a state-of-the-art 3D pose-estimation library (ROMP). A key step in the calculation of gait features from video is heel-strike detection; so, two methods for detecting these events were examined: manual annotation and automatic annotation using the ST-DBSCAN algorithm.

The initial correlation analysis between Zeno and video gait features calculated using 2D pose-estimation libraries highlighted that the automated ST-DBSCAN heel-strike method failed to detect short steps (Table 4, Fig. 3). Shuffling steps are not far enough apart spatially from the camera’s frontal view so the algorithm clusters many steps together, or misses them entirely if the step is very short and cannot be identified clearly in the joint trajectory data.

A 20-step cut-off was thus selected to facilitate a comparison of gait features for which both the manual and ST-DBSCAN methods were able to analyze roughly the same steps. This threshold was selected empirically for the data collection scenario used in this experiment and is likely to vary depending on the camera, as well as its angle and position with respect to the participant. In this investigation, the data collection set-up and selected 20-step threshold resulted in the exclusion of walks with a mean step length of 10.8 ± 5.4 cm, suggesting that the presented ST-DBSCAN method struggles with short, shuffling steps. However, the number of step or step length threshold at which the method fails will vary depending on the experimental set-up and should thus be determined empirically for future studies in different settings.

### A. Correlation of Zeno and Gait Features from 2D Pose-Estimation Libraries

As presented in Table 5, the Spearman’s rank correlation coefficient was significantly greater than 0 for most of the gait feature pairs compared. Some key observations can be made by comparing the ST-DBSCAN and manual annotation columns of Table 5. Specifically, it was observed that there was stronger correlation for the number of steps and cadence when the manual heel-strike annotation method was used. This is because the ST-DBSCAN algorithm uses the underlying joint trajectory to determine the timing and location of the heel-strikes, whereas during manual annotation the timing of heel-strikes is identified by viewing the video, while the location is determined by looking at the position of the joint trajectory at the time of the labelled heel-strike. In the case of number of steps and cadence, the timing of the heel strikes is the only important variable, and can be determined more accurately and consistently through manual annotation.

Conversely, the calculation of step width relies on the positions of the heel-strikes as well. The ST-DBSCAN algorithm requires that the underlying joint trajectory signal be sufficiently noise-free to facilitate detection of heel-strikes. Therefore, this method only identifies heel-strikes that are clearly captured by the joint trajectory obtained by the pose-estimation library. No heel-strikes will be detected when the joint trajectory is very noisy and more likely to be inaccurate. In comparison, manual annotation of heel-strikes was done directly from video, so it is possible that heel-strikes are selected at times where the underlying joint trajectory signal is noisy, resulting in less accurate estimations of step width.

From Table 5, it was also noted that the correlation between step time CV from video and step/swing time CV from the Zeno was poor across all pose-estimation libraries and directions when calculated using the ST-DBSCAN heel-strike annotation method, but moderate when manual annotation was used. These results highlight that at least moderate correlation can be achieved for these features between the two data collection methodologies, but the errors in detecting the timing of heel-strikes introduced by the ST-DBSCAN method are large enough to eliminate this correlation.

Due to the use of different units for distance measures, it is only possible to directly compare the number of steps and two temporal gait parameters calculated from video and from Zeno. Table C of the Supplement presents the mean difference, mean absolute difference, and percent difference between the Zeno and video features for the directly comparable features explored in this study (i.e., number of steps, cadence, and step time CV). This analysis further confirmed that the manual annotation method generally led to a lower percentage difference for the temporal measures.

Another key observation from Table C and Table 5 is that even a statistically significant positive correlation between Zeno and video features does not guarantee that the estimated parameters from video are accurate. For example, the correlation between step time CV calculated using manual heel-strike annotation and 2D pose-estimation libraries is moderate and statistically significantly greater than 0 (Table 5), but the percent difference between the values estimated by the two modalities ranges from 65 – 90% depending on the direction of walk (Table C). For this reason, values estimated from video cannot be directly compared to those collected using a Zeno instrumented walkway. Instead, it is recommended that trends in gait features be compared over time using the same data collection and processing methodology. Our correlation results indicate that data from video capture the same trends as an instrumented walkway for several key gait features, and could thus be used to track longitudinal changes in gait in individuals with PD.

#### 1) Comparison of 2D Pose-Estimation Libraries

As seen in Table 3, there are differences in the number of walks for which gait features were able to be calculated across the three pose-estimation libraries and two heel-strike annotation methods. All methods require that at least three steps be detected within the available ankle joint trajectory data. Therefore, the differences in the number of walks with successfully extracted video gait features for the manual annotation method can be directly attributed to the presence of the underlying joint trajectories. Based on the results presented in Table 3, it can be concluded that the OpenPose and Detectron libraries capture joint trajectories more consistently when the participant is walking away from the camera, while the AlphaPose library better tracks joint trajectories in walks towards the camera. Conversely, when the ST-DBSCAN heel-strike algorithm was used, gait features were successfully calculated in a total of 126 walks using Detectron, 115 using AlphaPose, and 101 when OpenPose was used to extract the joint trajectories. Notably, gait features were successfully extracted for more walking bouts towards the camera than for bouts away from the camera all pose-estimation libraries when using the ST-DBSCAN heel-strike detection method. Since this algorithm relies on the quality of the joint trajectories (rather than just their presence) to identify heel-strikes, the number of walks for which gait features were able to be calculated is a measure of how accurate and noise-free the underlying joint trajectories are when extracted by each pose-estimation library.

Comparing the correlations of Zeno and video gait features across the different 2D pose-estimation libraries, no consistent trends were observed across all gait feature pairs and directions of walk. However, there are significant fluctuations depending on the direction of the walking bout and by gait feature pair. For example, the difference in the strength of the correlation of step/stride width mean and CV varies less for walking bouts towards and away from the camera for the Detectron library than for the AlphaPose or OpenPose libraries. This difference in correlation strength is particularly pronounced when the ST-DBSCAN method is used. Furthermore, a similar difference in gait features calculated using the two footfall detection methods is noted when paired walks collected under different medication and DBS treatment states were analyzed. As observed in Table D of the Supplemental Material, gait features calculated using the manual footfall detection method were more commonly associated with expected significant differences between ON and OFF treatment states. For these reasons, it may be beneficial to use multiple pose-estimation libraries to calculate gait features and select the appropriate one based on the direction of movement, heel-strike annotation method, and gait feature of interest.

### B. Correlation of Zeno and Gait Features From 3D Pose-Estimation Library

As seen in Table 6, repeating the correlation analysis using gait features extracted using ROMP, a state-of-the-art 3D pose-estimation library, resulted in weaker correlations across all pairs of features. Notably, the gait features that relied on data in the depth dimension (such as gait speed/velocity and step length) had the lowest correlation coefficients across all pairs for the manual annotation method. This suggests that the ROMP library struggles to accurately track the position of joints in the anterior-posterior direction for the duration of each walking bout, leading to inaccurate calculations of video gait features that rely on data in this direction. This can be further confirmed by noting that the correlations between step/stride width (in the lateral direction) are stronger than those for step length (anterior-posterior direction) when manual annotation is used. This observation suggests that the underlying joint trajectory signal extracted using ROMP is noisy and inconsistent in its tracking of joints in the depth direction, which prevents meaningful use of this depth data. Overall, based on our experiments there is currently no advantage to using a state-of-the-art 3D pose-estimation library (ROMP) over a 2D one (AlphaPose, Detectron, or OpenPose) for calculation of gait features from video.

### C. Note on Video Data Collection Methodology

The videos used in this study were collected during clinical visits but were analyzed retrospectively. For this reason, changes in camera position and orientation were common as the data collection procedure was not standardized in this regard. However, as demonstrated by our results, the gait feature extraction methods used in this work are robust to such changes in video viewpoints, with moderate to strong correlations between video and Zeno features across many gait feature pairs. As camera position was not controlled or varied between pre-defined positions during data collection, it was not possible to do a robust analysis with respect to the correlations change with respect to camera viewpoints. However, previous work by our group comparing the correlation between gait features calculated from video and those obtained from a body-worn 3D motion capture system demonstrated that there was not a significant difference between cameras recording at different heights and angles [22]. These results are particularly promising when planning for the transition of this technology to non-clinical settings where differences between video recording methodologies will be common.

## V. Conclusion and Future Work

In this work, we examined the correlation between six pairs of gait features calculated from color video and a Zeno instrumented walkway. Feature pairs that only require the timing of heel-strikes are more strongly correlated between video and Zeno, while step/stride width mean and CV, which also require the spatial positions of the heel-strikes are only moderately correlated. In our experiments, we note that the ST-DBSCAN automated heel-strike detection algorithm leverages the underlying joint trajectory signal when identifying the timing of heel-strikes, and was thus similarly correlated to Zeno gait features as when manual annotation is used.

In our analyses, the automatic ST-DBSCAN heel-strike annotation method struggled to identify short, shuffling steps. Using an experimentally selected cut-off threshold of 20 steps for our dataset, walks with an average step length of 10.8 ± 5.4 cm (range: 4.8 – 19.2 cm), and representing less than 7% of all walking bouts were omitted from further analysis. This suggests that manual annotation is necessary to analyze gait of individuals with severe PD who have very short steps or experience freezing of gait. We also note that there were differences in the video recording methodology for this dataset, leading to differences in the viewpoint from which the videos were recorded. This suggests that the proposed method of calculating gait features from video is robust and shows promise as this work moves out of the clinic and is applied to videos collected in less controlled non-clinical environments.

Overall, the gait feature values calculated from video and Zeno cannot be used interchangeably as they were calculated using different methods and have different units (in the case of step/stride width). However, the gait features are significantly correlated when 2D pose-estimation libraries and appropriate heel-strike annotation methods are used. Our video-based gait analysis tool can thus be used for longitudinal monitoring of changes in gait in a PD population by comparing *trends* within features estimated by the video modality. This information can be used by clinicians to assess the impact of medication and DBS therapies and make appropriate adjustments to minimize gait impairments.

A limitation of this work is that gait features such as step length that rely on data in the anterior-posterior direction could not be calculated with the trajectories extracted using the 2D pose-estimation libraries. For this reason, ROMP, a state-of-the-art 3D pose-estimation library operating on monocular video was also explored as a means of extracting joint trajectories from video. However, the ROMP library struggled to accurately track the position of joints in the anterior-posterior direction for the duration of each walking bout, leading to inaccurate calculations of gait features that rely on data in this direction. For this reason, there is currently no advantage to using ROMP over the three 2D pose-estimation libraries investigated in this work.

Future work will seek to improve the estimation of 3D gait parameters from standard video. The features calculated in this work relied on the extraction of skeleton trajectories from video as a preliminary step. Instead, the estimation of gait features, including those that require information from the depth dimension such as gait speed and step length, could be incorporated directly into a machine learning model that operates on the input videos. This would allow for the development of an end-to-end framework that is finetuned for predicting more accurate gait features, rather than relying on general pose-estimation libraries to first extract joint trajectories.

## Supplementary Materials

Supplementary materials
