# Supplementary material for: Concurrent Validity of Zeno Instrumented Walkway and Video-Based Gait Features in Adults With Parkinson’s Disease
Source: IEEE J Transl Eng Health Med. 2022 Jun 3;10:2100511. doi: 10.1109/JTEHM.2022.3180231 (PMC9252334; doi:10.1109/JTEHM.2022.3180231)
Supplement: Supplementary materials [file supp1-3180231.pdf]

Supplementary Material

Concurrent validity of Zeno instrumented walkway and video-based gait features in adults with Parkinson’s disease

AlphaPose

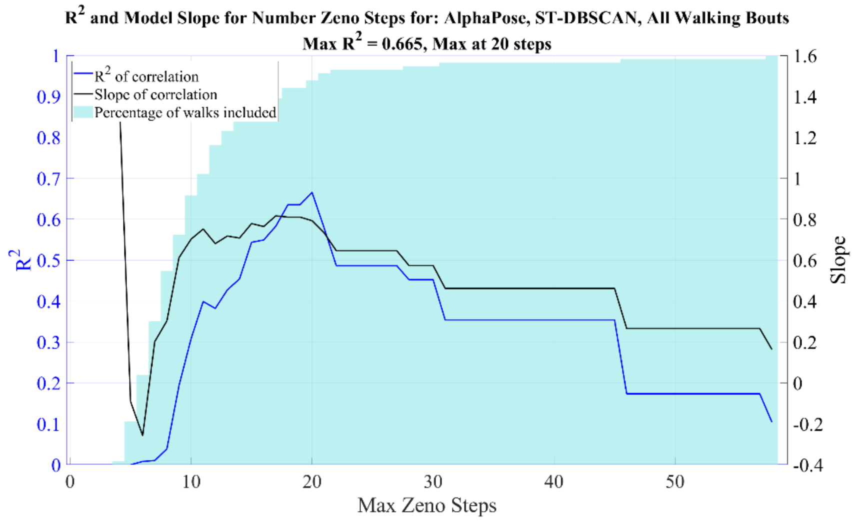

Detectron

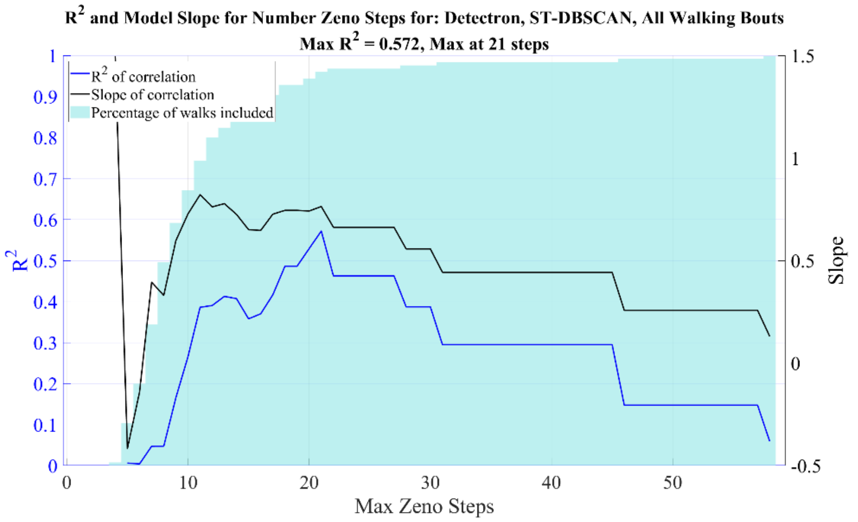

OpenPose

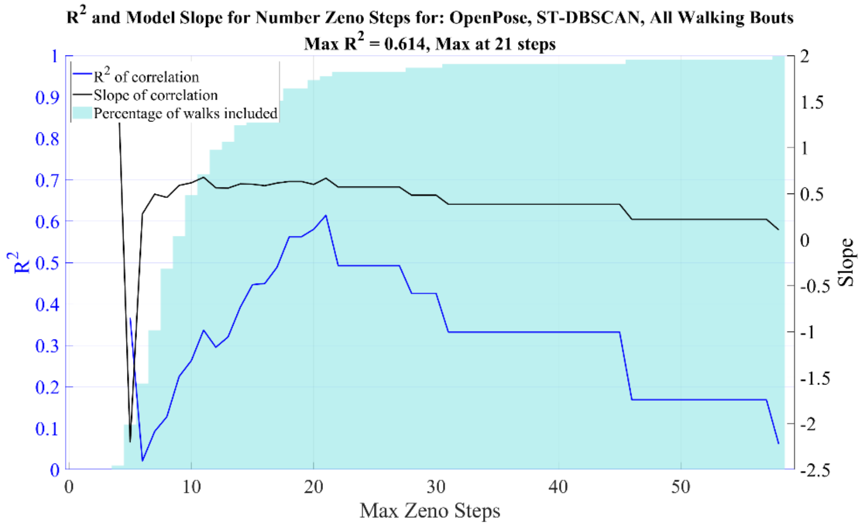

Fig. A. Model slope and R^2 for correlation between number of steps detected by Zeno and number of steps detected using the ST-DBSCAN method on video.

TABLE A. P-VALUES FOR D'AGOSTINO-PEARSON NORMALITY TEST OF ZENO AND VIDEO FEATURES EXTRACTED FROM 2D POSE-ESTIMATION LIBRARIES

				ST-DBSCAN						Manual					
				Away from Camera		Towards Camera		Both		Away from Camera		Towards Camera		Both	
	**Video gait feature**	**Zeno gait feature**	**Detector**	**Video p**	**Zeno p**	**Video p**	**Zeno p**	**Video p**	**Zeno p**	**Video p**	**Zeno p**	**Video p**	**Zeno p**	**Video p**	**Zeno p**
All Steps	Number of steps	Number of steps	AlphaPose	<.001	**.194**	<.001	<.001	<.001	<.001	<.001	<.001	<.001	<.001	<.001	<.001
			Detectron	<.001	.043	<.001	<.001	<.001	<.001	<.001	<.001	<.001	<.001	<.001	<.001
			OpenPose	<.001	**.161**	<.001	<.001	<.001	<.001	<.001	<.001	<.001	<.001	<.001	<.001
	Cadence	Cadence	AlphaPose	<.001	<.001	<.001	.002	<.001	<.001	<.001	<.001	<.001	<.001	<.001	<.001
			Detectron	<.001	<.001	<.001	.028	<.001	.003	<.001	<.001	<.001	<.001	<.001	<.001
			OpenPose	.048	**.233**	<.001	**.077**	<.001	**.067**	<.001	<.001	<.001	<.001	<.001	<.001
	Step width – mean	Stride width – mean	AlphaPose	**.102**	<.001	<.001	.002	<.001	<.001	.023	<.001	<.001	.006	<.001	<.001
			Detectron	<.001	<.001	<.001	<.001	<.001	<.001	<.001	.002	<.001	<.001	<.001	<.001
			OpenPose	**.171**	<.001	<.001	<.001	<.001	<.001	**.054**	<.001	<.001	.010	<.001	<.001
	Step width - CV	Stride width – CV	AlphaPose	.002	**.297**	<.001	.007	<.001	.011	.013	**.214**	<.001	**.148**	<.001	**.105**
			Detectron	.002	<.001	<.001	.027	<.001	<.001	.020	**.205**	<.001	**.272**	<.001	**.066**
			OpenPose	.003	**.509**	<.001	.020	<.001	.019	.014	<.001	<.001	**.194**	<.001	<.001
	Step time - CV	Step time – CV	AlphaPose	<.001	.003	<.001	<.001	<.001	<.001	<.001	<.001	<.001	<.001	<.001	<.001
			Detectron	<.001	.028	<.001	<.001	<.001	<.001	<.001	<.001	<.001	<.001	<.001	<.001
			OpenPose	<.001	<.001	<.001	<.001	<.001	<.001	<.001	<.001	<.001	<.001	<.001	<.001
	Step time - CV	Swing time – CV	AlphaPose	<.001	.003	<.001	<.001	<.001	<.001	<.001	<.001	<.001	<.001	<.001	<.001
			Detectron	<.001	.028	<.001	<.001	<.001	<.001	<.001	<.001	<.001	<.001	<.001	<.001
			OpenPose	<.001	<.001	<.001	<.001	<.001	<.001	<.001	<.001	<.001	<.001	<.001	<.001
20 Steps	Number of steps	Number of steps	AlphaPose	**.139**	**.220**	<.001	<.001	<.001	<.001	.041	**.061**	.002	<.001	<.001	<.001
			Detectron	.019	.034	<.001	<.001	<.001	<.001	.041	**.061**	.002	<.001	<.001	.001
			OpenPose	**.227**	**.195**	<.001	.031	<.001	.011	**.053**	**.068**	.002	<.001	<.001	.001
	Cadence	Cadence	AlphaPose	<.001	.002	<.001	.008	<.001	.005	<.001	<.001	<.001	<.001	<.001	<.001
			Detectron	<.001	<.001	<.001	**.052**	<.001	.008	<.001	<.001	<.001	<.001	<.001	<.001
			OpenPose	.024	**.151**	<.001	.028	<.001	.040	<.001	<.001	<.001	<.001	<.001	<.001
	Step width – mean	Stride width – mean	AlphaPose	**.067**	<.001	<.001	<.001	<.001	<.001	.008	<.001	<.001	.001	<.001	<.001
			Detectron	<.001	<.001	<.001	<.001	<.001	<.001	<.001	<.001	<.001	<.001	<.001	<.001
			OpenPose	**.100**	<.001	<.001	<.001	<.001	<.001	.021	<.001	<.001	.005	<.001	<.001
	Step width - CV	Stride width – CV	AlphaPose	.003	**.297**	<.001	.006	<.001	.012	.020	**.137**	<.001	**.288**	<.001	**.209**
			Detectron	.003	<.001	<.001	.013	<.001	<.001	.031	**.170**	<.001	**.471**	<.001	**.109**
			OpenPose	.004	**.484**	<.001	.008	<.001	.009	.022	<.001	<.001	**.298**	<.001	<.001
	Step time - CV	Step time – CV	AlphaPose	<.001	**.566**	<.001	.002	<.001	.021	<.001	<.001	<.001	.003	<.001	<.001
			Detectron	<.001	**.083**	<.001	.002	<.001	<.001	<.001	<.001	<.001	.013	<.001	<.001
			OpenPose	<.001	**.308**	<.001	<.001	<.001	.005	<.001	<.001	<.001	.007	<.001	<.001
	Step time - CV	Swing time – CV	AlphaPose	<.001	**.566**	<.001	.002	<.001	.021	<.001	<.001	<.001	.003	<.001	<.001
			Detectron	<.001	**.083**	<.001	.002	<.001	<.001	<.001	<.001	<.001	.013	<.001	<.001
			OpenPose	<.001	**.308**	<.001	<.001	<.001	.005	<.001	<.001	<.001	.007	<.001	<.001

*H_0: Samples come from a normal distribution. Bolded values represent features set for which the null hypothesis was not rejected (ie. normally distributed feature sets)

Table B. P-Values for D'Agostino-Pearson Normality test of Zeno and Video Features Extracted from the ROMP 3D Pose-Estimation Library

			**ST-DBSCAN**						**Manual**					
			**Away from Camera**		**Towards Camera**		**Both**		**Away from Camera**		**Towards Camera**		**Both**	
	**Video gait feature**	**Zeno gait feature**	**Video p**	**Zeno p**	**Video p**	**Zeno p**	**Video p**	**Zeno p**	**Video p**	**Zeno p**	**Video p**	**Zeno p**	**Video p**	**Zeno p**
All Steps	Number of Steps	Number of Steps	<.001	<.001	<.001	<.001	<.001	<.001	<.001	<.001	<.001	<.001	<.001	<.001
	Cadence	Cadence	**.051**	.012	**.707**	**.149**	**.537**	.004	.006	.014	**.062**	**.139**	.006	.003
	Speed	Velocity	<.001	<.001	<.001	<.001	<.001	<.001	<.001	<.001	<.001	<.001	<.001	<.001
	Step Width - mean	Stride Width - mean	<.001	**.240**	.004	**.476**	<.001	**.190**	**.067**	**.178**	**.768**	**.626**	**.366**	**.218**
	Step Length - mean	Step Length - mean	<.001	**.170**	<.001	**.128**	<.001	.032	<.001	**.187**	.001	**.094**	<.001	.029
	Step Width - CV	Stride Width - CV	**.069**	.001	**.699**	<.001	**.706**	<.001	.047	.003	<.001	<.001	<.001	<.001
	Step Time - CV	Step Time - CV	**.335**	<.001	**.964**	<.001	**.779**	<.001	<.001	<.001	<.001	<.001	<.001	<.001
	Step Time - CV	Swing Time - CV	**.335**	<.001	**.964**	<.001	**.779**	<.001	<.001	<.001	<.001	<.001	<.001	<.001
20 Steps	Number of Steps	Number of Steps	**.059**	**.120**	<.001	<.001	<.001	<.001	**.500**	**.150**	<.001	<.001	<.001	<.001
	Cadence	Cadence	.022	**.194**	**.266**	.029	**.287**	.044	**.253**	**.199**	.001	.021	.032	.035
	Speed	Velocity	<.001	<.001	.002	<.001	<.001	<.001	<.001	.007	<.001	<.001	<.001	<.001
	Step Width - mean	Stride Width - mean	<.001	**.347**	.007	**.540**	<.001	**.288**	**.063**	**.286**	**.581**	**.706**	**.225**	**.343**
	Step Length - mean	Step Length - mean	<.001	**.278**	<.001	**.431**	<.001	**.142**	**.395**	**.278**	.002	**.468**	.005	**.136**
	Step Width - CV	Stride Width - CV	.047	.002	**.812**	<.001	**.563**	<.001	**.080**	.007	<.001	<.001	<.001	<.001
	Step Time - CV	Step Time - CV	**.470**	<.001	**.924**	<.001	**.873**	<.001	<.001	<.001	<.001	<.001	<.001	<.001
	Step Time - CV	Swing Time - CV	**.470**	<.001	**.924**	<.001	**.873**	<.001	<.001	<.001	<.001	<.001	<.001	<.001

*H_0: Samples come from a normal distribution. Bolded values represent features set for which the null hypothesis was not rejected (ie. normally distributed feature sets)

TABLE C. MEAN DIFFERENCE, ABSOLUTE MEAN DIFFERENCE, AND MEAN PERCENT DIFFERENCE WITH RESPECT TO ZENO FOR THREE PAIRED GAIT FEATURES FROM VIDEO AND ZENO FOR WALKING BOUTS WITH A MAXIMUM OF 20 STEPS

			ST-DBSCAN									Manual								
			Away from Camera			Towards Camera			Both			Away from Camera			Towards Camera			Both		
**Video gait feature**	**Zeno gait feature**	**Detector**	**Mean diff.**	**Mean absolute diff.**	**Percent different from Zeno**	**Mean diff.**	**Mean absolute diff.**	**Percent different from Zeno**	**Mean diff.**	**Mean absolute diff.**	**Percent different from Zeno**	**Mean diff.**	**Mean absolute diff.**	**Percent different from Zeno**	**Mean diff.**	**Mean absolute diff.**	**Percent different from Zeno**	**Mean diff.**	**Mean absolute diff.**	**Percent different from Zeno**
Number of steps	Number of steps	AlphaPose	0.48	1.57	16.97	1.13	1.85	21.06	0.85	1.73	19.30	1.25	1.65	17.82	2.42	2.60	27.84	1.80	2.10	22.52
		Detectron	1.21	2.34	23.72	0.90	1.77	20.32	1.05	2.05	22.00	1.25	1.65	17.82	2.40	2.58	27.68	1.79	2.09	22.44
		OpenPose	1.92	2.25	20.11	1.58	2.19	23.21	1.71	2.21	22.03	1.27	1.68	18.09	2.43	2.58	27.68	1.82	2.11	22.63
		ROMP	-1.91	4.36	48.52	-2.44	4.28	47.05	-2.19	4.32	47.74	0.15	1.90	21.62	0.60	1.87	20.32	0.40	1.88	20.88
Cadence	Cadence	AlphaPose	-6.58	7.42	7.66	5.86	8.88	8.68	0.51	8.25	8.24	-3.45	5.09	5.32	-1.05	4.55	4.53	-2.33	4.84	4.95
		Detectron	-6.37	9.01	9.15	7.41	10.15	9.91	0.64	9.59	9.54	-3.45	5.09	5.32	-1.02	4.59	4.56	-2.31	4.85	4.97
		OpenPose	-6.85	8.01	8.96	5.39	9.08	9.05	0.75	8.67	9.02	-3.58	5.23	5.47	-0.92	4.49	4.48	-2.32	4.88	5.00
		ROMP	-38.26	42.66	44.44	-10.52	30.08	31.75	-23.50	35.97	37.69	-2.84	6.25	6.85	2.49	4.33	4.15	0.17	5.17	5.32
Step time - CV	Step time – CV	AlphaPose	-0.20	0.23	400.21	-0.07	0.24	349.85	-0.12	0.23	371.50	-0.02	0.07	88.85	0.13	0.17	66.88	0.05	0.12	78.56
		Detectron	-0.21	0.23	384.22	-0.05	0.29	402.96	-0.13	0.26	393.75	-0.02	0.07	88.85	0.13	0.17	67.02	0.05	0.12	78.62
		OpenPose	-0.22	0.26	426.55	-0.03	0.27	294.72	-0.10	0.27	344.68	-0.02	0.07	89.88	0.13	0.17	64.96	0.05	0.12	78.10
		ROMP	-0.55	0.57	1019.48	-0.48	0.57	1039.37	-0.51	0.57	1030.06	-0.02	0.08	106.49	0.01	0.11	126.49	0.00	0.10	117.70

*Note: The measurements from Zeno were taken as ground truth and the percent difference was thus computed as |video feature – Zeno feature| / (Zeno feature)

Supplemental Analysis Under Different Treatment Conditions:

A further analysis was performed to evaluate whether gait features extracted from the Zeno-PKMAS and the video, respectively, could be used to differentiate between walks of individuals with PD under different treatment conditions. For this analysis, the subset of walks which were collected during clinical visits in which the participant was evaluated in both ON and OFF treatment states was used. Walks in the OFF treatment state were recorded at least 12 hours after the participant had taken their last prescribed dose of dopaminergic medication and when their subthalamic nucleus deep brain stimulation (DBS) device was turned off. Conversely, walks in the ON state were recorded 45 – 60 minutes after the participant took their dopaminergic medication and with their DBS device turned on. Walks were paired by participant and clinical visits, with two recorded walks for each visit (one in each of the ON and OFF states). A total of 21 clinical visits (42 walking bouts) from 13 participants were included in the analysis. As in the analysis in the main manuscript, bouts with less than 3 and more than 20 detected steps were omitted from subsequent analysis.

A Wilcoxon signed-rank test (a non-parametric version of the paired t-test) was used to evaluate whether there was a significant difference between the gait features recorded in the ON and OFF states when using the Zeno and video-based gait features. Table D, below, presents the p-values for the null hypothesis that the features in the ON and OFF state come from the same distribution. Lower p-values indicate that there is more likely to be a difference between the specified gait feature when measured in the ON and OFF states during the same clinical visit. From a clinical perspective, it is expected that there will be a significant difference in each gait feature in the ON and OFF treatment states [1].

TABLE D
P-VALUES FOR WILCOXON SIGNED-RANK TEST BETWEEN ON AND OFF STATES FOR PAIRED WALKS BY GAIT FEATURE, POSE-ESTIMATION LIBRARY, DIRECTION OF WALK, AND FOOTFALL DETECTION METHOD FOR BOUTS WITH A MAXIMUM OF 20 STEPS

			**ST-DBSCAN**						**Manual**					
			**Away from Camera**		**Towards Camera**		**Both**		**Away from Camera**		**Towards Camera**		**Both**	
**Video gait feature**	**Zeno gait feature**	**Detector**	**Video Feature *p***	**Zeno Feature *p***	**Video Feature *p***	**Zeno Feature *p***	**Video Feature *p***	**Zeno Feature *p***	**Video Feature *p***	**Zeno Feature *p***	**Video Feature *p***	**Zeno Feature *p***	**Video Feature *p***	**Zeno Feature *p***
Number of steps	Number of steps	AlphaPose	.326	**.006**	**.013**	**.003**	**.020**	**<.001**	**.005**	**<.001**	**.007**	**.004**	**<.001**	**<.001**
		Detectron	.068	**.001**	**.017**	**.003**	**.002**	**<.001**	**.005**	**<.001**	**.006**	**.004**	**<.001**	**<.001**
		OpenPose	.592	**.007**	**.021**	**.004**	**.046**	**<.001**	**.009**	**.001**	**.006**	**.004**	**<.001**	**<.001**
Cadence	Cadence	AlphaPose	**.011**	.626	.284	**.005**	**.012**	**.020**	.073	.709	**.049**	.091	**.008**	.131
		Detectron	.468	.417	**.031**	**.027**	**.046**	**.028**	.073	.709	**.049**	.091	**.008**	.131
		OpenPose	**<.001**	.850	**.021**	.065	**<.001**	.133	**.016**	.417	**.049**	.091	**.002**	.059
Step width – mean	Stride width – mean	AlphaPose	.296	**.013**	.145	**.027**	.799	**<.001**	.879	**.014**	.068	**.011**	.270	**<.001**
		Detectron	.265	**.027**	.174	**.035**	.819	**.002**	.860	**.014**	**.035**	**.011**	.280	**<.001**
		OpenPose	**.012**	.064	.175	.065	.412	**.008**	.442	**.027**	**.020**	**.011**	.588	**<.001**
Step width - CV	Stride width – CV	AlphaPose	**<.001**	**<.001**	.051	**.017**	**<.001**	**<.001**	**.016**	**<.001**	.058	**.001**	**.001**	**<.001**
		Detectron	**.003**	**<.001**	.089	**.011**	**.001**	**<.001**	**.001**	**<.001**	**.049**	**.001**	**<.001**	**<.001**
		OpenPose	**.005**	**<.001**	**.004**	**.029**	**<.001**	**<.001**	.181	**<.001**	**.009**	**.001**	**.007**	**<.001**
Step time - CV	Step time – CV	AlphaPose	.068	.463	.548	.071	.367	.066	**<.001**	.215	**.013**	.091	**<.001**	**.030**
		Detectron	.246	.287	.431	.145	.896	.074	**<.001**	.215	**.013**	.091	**<.001**	**.030**
		OpenPose	.301	.328	.900	.274	.387	.156	**.001**	.287	**.013**	.091	**<.001**	**.044**
Step time - CV	Swing time – CV	AlphaPose	.068	.119	.548	.174	.367	**.040**	**<.001**	**.014**	**.013**	.068	**<.001**	**.003**
		Detectron	.246	**.024**	.431	.174	.896	**.011**	**<.001**	**.014**	**.013**	.068	**<.001**	**.003**
		OpenPose	.301	.052	.900	.175	.387	**.019**	**.001**	**.024**	**.013**	.068	**<.001**	**.004**

Several observations can be made from Table D. Firstly, it can be seen that gait features from Zeno are significantly different in ON and OFF states more often than the analogous video-based gait feature when the automatic ST-DBSCAN footfall detection method is used. Conversely, when the footfalls are manually annotated, the differences between ON and OFF states are more likely to be significant, particularly for the comparisons comparing step time CV. This observation is consistent with the Spearman's correlation results presented in Table V where the correlations between the step time CV gait feature were only significant when the manual footfall detection method was used. In general, the gait features for which there are most consistent differences in ON and OFF states are the number of steps, and the step/stride width CV.

It is also noteworthy that there is more likely to be a difference between ON and OFF states when walking bouts in which the participant is walking towards and away from the camera are included. This suggests that the small size of the dataset when using only walking bouts towards or only away from the camera is insufficient to thoroughly analyze the difference between ON and OFF states. Future work is needed on a larger dataset with paired ON and OFF states to better understand whether there are significant differences in gait features between ON and OFF states.

Reference:

[1] R. Bouça-Machado *et al.*, “Gait kinematic parameters in Parkinson’s disease: a systematic review,” *J. Parkinsons. Dis.*, vol. 10, no. 3, pp. 843–853, 2020.
